# Experimental and Numerical Investigations of Cement Bonding Properties at Elevated Temperatures—The Effect of Sample Cooling

**DOI:** 10.3390/ma15144955

**Published:** 2022-07-16

**Authors:** Ionut Lambrescu, Catalin Teodoriu

**Affiliations:** 1 Department Infromatica, Tehnologia Informatiei, Matematica si Fizica (ITIMF), Petroleum-Gas University Ploiesti, 100680 Ploiesti, Romania; ilambrescu@upg-ploiesti.ro; 2 Mewbourne School of Petroleum and Geological Engineering, The University of Oklahoma, Norman, OK 73019, USA

**Keywords:** well construction, well integrity, well cementing, elevated temperature, post-failure analysis, interfacial bonding strength of cement

## Abstract

Well integrity is currently defined through the concept of well barriers, in which one or more barriers are used to prevent unwanted fluid flow. Many papers have highlighted that the casing–cement interfacial bonding is critical for well integrity, but many discrepancies between laboratory experiments and field data have been noticed. The use of finite element analysis is now established as an alternative to complex in situ tests, but these simulations are sensitive to the input parameters, which results in many discrepancies across published works. Currently, the cohesive zone material (CZM) method is considered to offer good results if the correct parameters are selected or experimentally determined. The novelty of this paper lies in the development of a better workflow that enables the simulation of three processes that are acting on the laboratory-scale casing–cement system: temperature changes, debonding, and post-debonding behavior. The aim of this paper is to fully understand the debonding process within laboratory-scale samples, and thus to eventually enable upscaling in the near future. The paper presents a new workflow generated using FEM that allows us to determine the contact stresses at the casing–cement interface during temperature changes at the moment of debonding and post-debonding. The results presented within this paper show that temperature samples tested according to the push-down setup will provide similar interfacial bonding shear strength values; however, post-debonding, there is a remaining frictional force slightly higher than that of the room-temperature samples. In this case, the results are within a 5% error of the average field data, which is slightly higher than in our previous experiments, where only room temperature data were considered. A major outcome of our paper is the demonstration of the existence of friction forces after debonding, which are a result of radial stresses induced during the debonding process.

## 1. Introduction

Wellbore integrity studies have revealed that the main failure during the life of the well occurs within the casing–cement system. The main function of the wellbore cement is to seal the annular space between the casing and the formation through mechanical and hydraulic seals [1]. Therefore, the long-term stability of the cement properties, as well as the strong bond between the cement and the casing, is critical for our further understanding and modeling of well integrity [1,2,3,4]. Casing–cement bonding is considered to be key for long-lasting well integrity; thus, our understanding of the debonding and post-debonding behavior of the casing–cement system becomes very important. If debonding between the casing and the cement takes place in a well, the system’s integrity may be affected mechanically (by loss of support, resulting in relative motion between the casing and the cement) and/or hydraulically (by forming a micro-annulus that will create a pathway for leakage). The latter is more dangerous, as it will result in unexpected fluid migration to the surface, thus resulting in environmental pollution and accidents.

Evans [5] is acknowledged as having produced the first report on casing–cement bonding investigations, defining the properties of bonding strength and hydraulic bonding strength. Teodoriu et al. [6] refined the cement bonding properties by the introduction of three different interfacial cement strengths, whereas the interfacial bonding shear strength (IBSS) is the equivalent to Evans’s bonding strength and measures the strength of the adherence (or bonding) between the casing and the cement. Geothermal wells are considered to be highly impacted by casing–cement shear bonding, which is induced by axial expansion/contraction of the casing relative to the cement. [7,8]. The major limitation of incorporating casing–cement bonding into well integrity studies is the limited access to accurate information about IBSS, resulting in extreme difficulties for finite element study [9]. Because the majority of the data published on IBSS focus on short-term investigations, such as 8, 24, or 72 h studies, long-term well integrity investigations using finite element analysis are more difficult. Long-term IBSS may be constant if curing conditions are unchanging [6].

IBSS testing consists of two methods: the push-out method and rotational disk method [10]. The modified Brazilian test was proposed for evaluating the mixed-mode interfacial strength by some authors [11]. The push-out method is the most frequently used setup, being based on Evans’s [5] initial experimental work; however, there are several variations of the approach, with the cement being inside a pipe or the cement filling the annular space [6,12,13,14,15,16,17,18,19]. 

In a previous paper, Lambrescu et al. [10] proposed a verification of the CZM method for modelling experimental tests that measure IBSS, demonstrating a strong correlation between experimental and numerical solutions. One question that arose from the previous work was how the elevated temperature testing should be performed in order to achieve good experimental results, but also to enable the safe handling of samples (cooling at room temperature). Therefore, the question investigated in this paper is whether the heating and cooling of the IBSS samples, as per [6,10], will have a direct impact on the results. To solve this problem, we use experimental data and finite element modeling that follows the same approach as presented in [10]. Additionally, we will introduce a post-debonding investigation methodology, which will enable us to understand the reason for the existence of a post-debonding force (we will call this F2, or frictional post-debonding force) which moves the cement. 

Jian et al. [20] documented that CZM is the best solution for modelling casing–cement debonding and showed that it can be used for both shear and tension cases. Yan et al. [21] developed a numerical and experimental methodology for modelling the debonding of the cementing interface during perforation jobs. Although Yan et al. [21,22] used ANSYS as a numerical simulation environment, they came to the conclusion that the CZM method may not fully describe the dynamical simulation they proposed; hence, they used simple casing–cement bonding models. 

Feng et al. [23] proposed a 3D numerical model for evaluating casing–cement debonding. However, 3D models can consume computing power and slow down some analyses. However, Feng et al. [23] also showed that the CZM method is the best way to model the casing–cement debonding process.

Wise et al. [24] used finite element simulation to identify the wellbore location, where debonding may occur, by quantification of micro-annuli gap. They proposed using the softening traction–separation law known as the CZM method, similarly to [25].

Although the CZM method has been widely used to simulate casing–cement contact and, thus, its failure mode, few papers have focused on the simulation of laboratory experiments, the investigation of which is the novelty of this paper. Furthermore, the post-debonding behavior observed during experimental testing has not been fully investigated to date, and it is proposed herein.

This paper seeks to demonstrate the following:

- The cooling of the samples has little to no effect on the IBSS results, but it will affect the post-debonding behavior of the cement–casing system.
- A new post-debonding model of the casing–cement system using friction bonding elements is proposed.
- A post-debonding friction force between the casing and the cement exists and will affect the relative motion between the casing and the cement.

The results of this paper will enable the further optimization of the experimental testing of the debonding process and will open a new direction for research on the post-debonding behavior of casing–cement systems.

## 2. Experimental Approach

The experimental approach used for this paper follows the same steps and material preparation as shown in [6,10]. Therefore, the sample preparation and experimental setup are not presented in detail in this paper. We will only highlight the differences from previous experiments.

### 2.1. Sample Preparation for Experimental Testing

Class H neat cement was mixed following the current cement standards [26,27]. The sample preparation, curing, and testing was planned according to the methodology developed by Teodoriu et al. [6]. The cement used was commercial class H cement, as per [28]. The metal cylinders, fabricated from 304 stainless steel, were cleaned and polished with 400-grid sandpaper prior to the cement pouring. All samples used for this paper were cured at room temperature and elevated (75 °C) temperature under atmospheric pressure. The samples were cured for 1, 3, and 7 days. The elevated-temperature samples were first allowed to slowly cool down to room temperature, then they were tested. 

### 2.2. Experimental Setup

The same experimental equipment and testing methodology as those presented in [6] were used. The cell (mold) sizes and geometry were presented in detail in [6,10]. Table 1 summarizes the geometry of the shear bonding cell. The values for length, outer diameter (OD), and inner diameter (ID) were average values based on a minimum of 4 measurements. These values were used for the numerical study of—as well as to calculate—the IBSS from the experimental tests. Equation (1) shows the relation between the experimentally measured force and the calculated IBSS.

The interfacial bonding shear strength (measured in MPa) is calculated as:

(1) $$\sigma =\frac{F_{max}}{2\pi *ID_{A}*CL}$$

where *F_max_* is the maximum recorded force, N;

*ID_A_* is the inner diameter of the cell, m;

*CL* is the interfacial bonding shear strength cell length, m.

**Figure 1:**
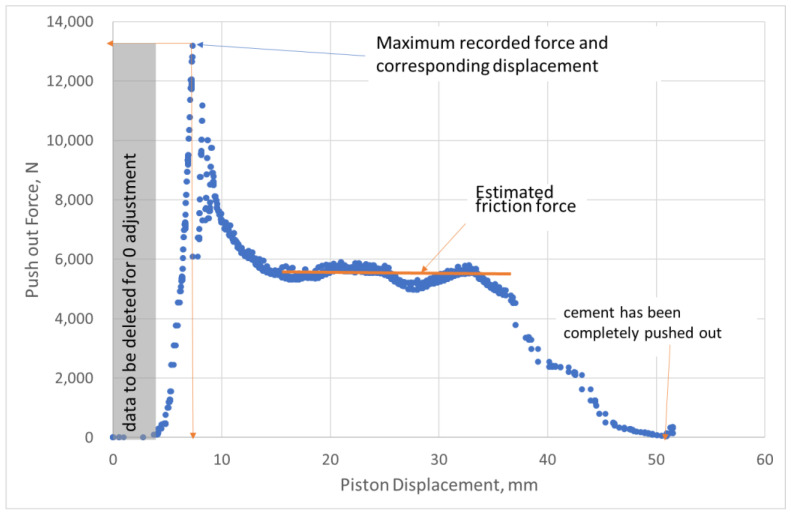
Generic push-out experiment showing the force as a function of piston displacement, modified after [10].

An example of the piston force versus piston displacement is shown in Figure 1. The maximum force, its corresponding relative displacement, and the maximum initial post-debonding force are retrieved from each graph.

Table 2 shows the calculated interfacial bonding shear strength for room-temperature and elevated-temperature samples after 1, 3, and 7 days. A minimum of 3 samples were used to calculate the presented values. 

## 3. The Finite Element Simulation

**Figure 2:**
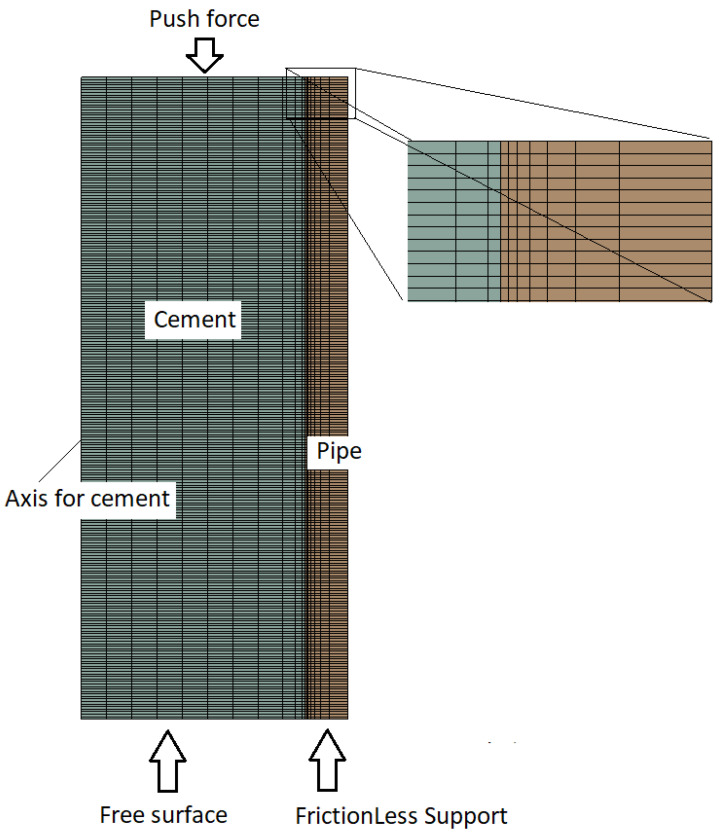
Model and meshing structure used for this study.

The ANSYS 2021 R2 version was used to perform the finite element simulation. The same axisymmetric model as that presented in [10] was used. The model and its mesh are shown in Figure 2.

### 3.1. Model Formulation and Contact Zones

Nonlinear mechanical quadratic axisymmetric elements with three or four edges were used for the whole cell in order to obtain better results. Again, this model requires two contact zones: the first zone is needed at the interface between the piston and the cement, which is used to estimate the force required to de-bond the cement and, for these investigations, is the main contact zone between the cement and the cylinder inside the wall [29,30].

The contact between the cement and the internal face of the pipe was modeled as bonded, followed by the application of a cohesive zone material (CZM). For the CZM, we chose the bilinear model, Mode II debonding, as in [10].

Once again, the parameters set out in the engineering data section in ANSYS are [22]:

- *T_t_^max^*—maximum equivalent tangential contact debonding (the value of this parameter will be extracted from the experimental work).
- δ_t_*—tangential slip at the completion of debonding (this value will also be extracted from the experimental data).
- Artificial damping coefficient—according to [22], this parameter is used for convergence purposes, and, normally, it is related to the incremental time step used for the simulation.

### 3.2. Load Application and Boundary Definition

The load application sequence for the elevated-temperature samples was modified from a previous paper [10] to assess the effect of temperature. The model initial temperature was 75 °C. Then, a slow cooling to about 30 °C was induced. The stresses resulting from cooling were mapped and used to conduct the second load application: push-out force. As before, the stainless steel cylinder was supported on the bottom face. A ramped load sequence was used for the push-out load sequence for both the room-temperature and elevated-temperature cases. 

The mechanical properties used for the cement and steel are shown in Table 3. Although several values have been used to match the results, Table 3 shows the final validated results. Thus, a sensitivity analysis is not shown in this paper. As a novelty, we also introduced the friction between the cement and the pipe after the debonding process. In this case, we varied the friction coefficient from 0.1 to about 0.8 to identify the best fit with the experimental data. 

### 3.3. Failure Mode Definition

The failure mode of the debonding is the same as that proposed by Lambrescu et al. [10]. Basically, we increased the force to about 125% of the experimentally observed force, after which we analyzed the simulation log files and observed the discontinuities of the various plots, such as axial displacement, contact status, reaction force at the piston face, and sliding distance. 

**Figure 3:**
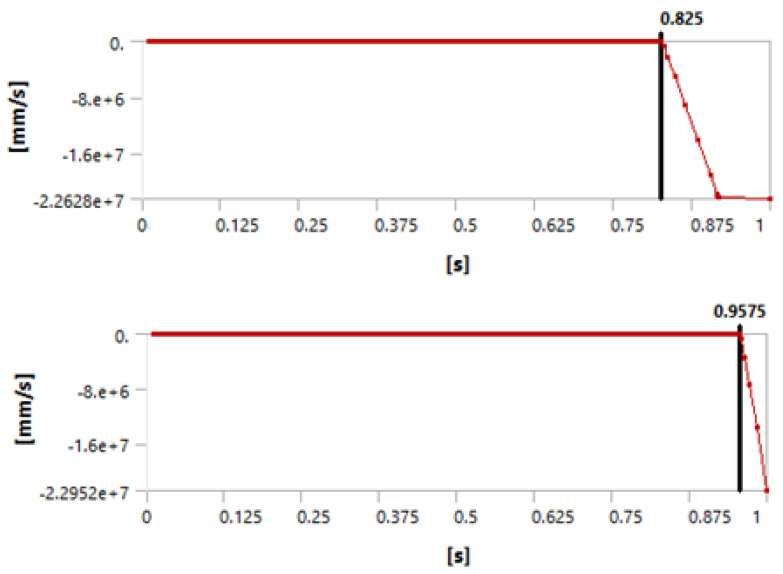
Debonding moment observed in the axial velocity plot showing an abrupt change in velocity. The (**top**) graph shows the plot for 1 day of curing and the (**bottom**) graph for 3 days of curing.

**Figure 4:**
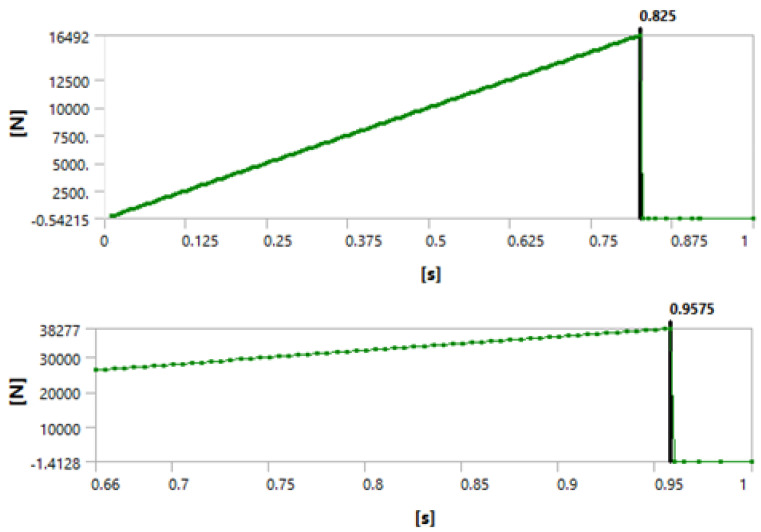
Debonding moment observed in the force reaction plot showing an abrupt change. The (**top**) graph shows the plot for 1 day of curing and the (**bottom**) graph for 3 days of curing.

**Figure 5:**
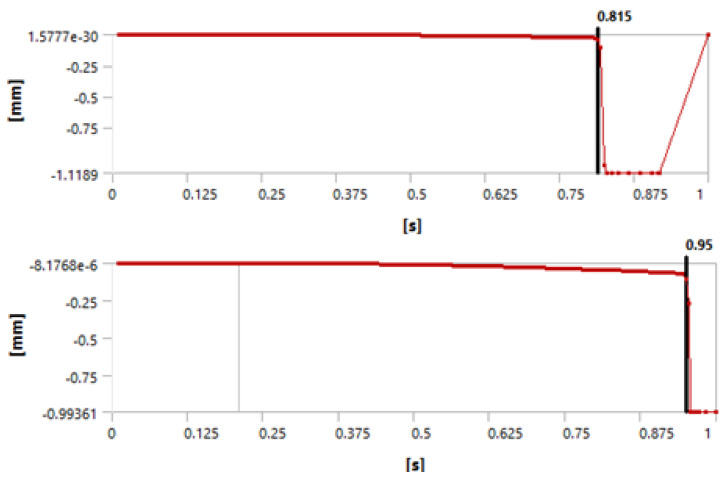
Debonding moment observed in the contact sliding distance plot showing an abrupt change in sliding distance. The (**top**) graph shows the plot for 1 day of curing and the (**bottom**) graph for 3 days of curing.

Figure 3, Figure 4 and Figure 5 show that we observed similar behavior in the room-temperature and elevated-temperature debonding processes, confirming once again that the CZM model used for numerical solutions is adequate.

Again, we consider four different methods of defining the time when the debonding process starts. A detailed discussion of this failure detection was published by Lambrescu et al. [10].

## 4. Results

**Figure 6:**
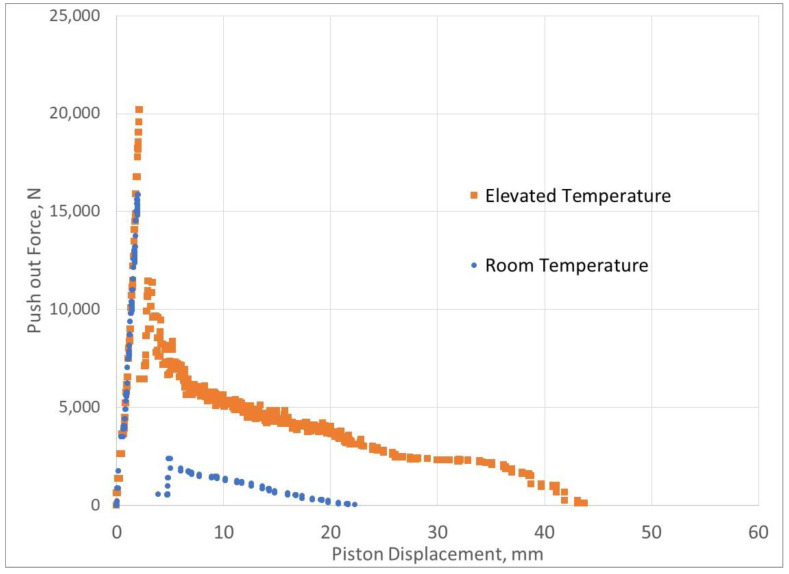
Push-out force vs. piston displacement for samples cured for 1 day at room temperature and elevated temperature.

**Figure 7:**
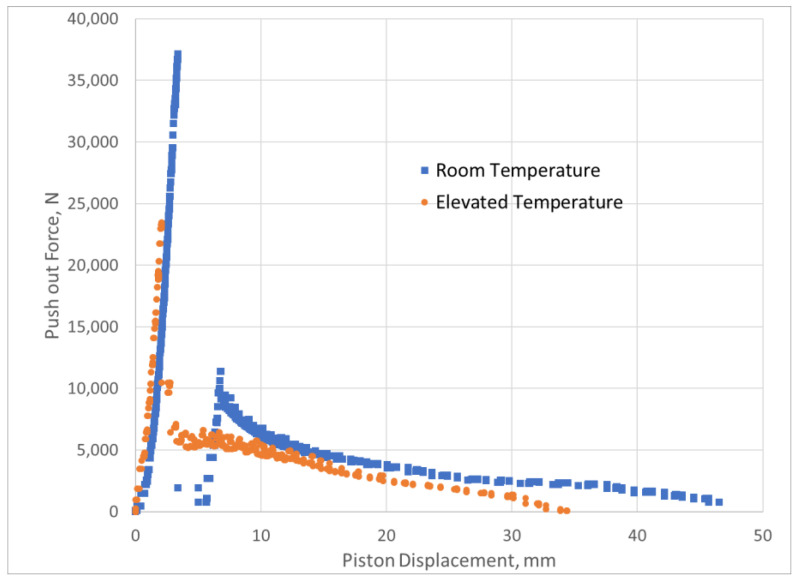
Push-out force vs. piston displacement for samples cured for 3 days at room and elevated temperature.

Figure 6 and Figure 7 show the force versus displacement curves from the experimental tests for the samples cured for 1 day and 3 days at room temperature and elevated temperature. Note that the push-out force for the elevated-temperature tests after 3 days of curing is lower than the force for the room-temperature tests. This was described by [6] and is attributed to the fact that elevated temperatures accelerate the hydration process, resulting in a weaker bond to the metallic pipe.

The slow decrease in the force with increasing piston displacement is normal, as the cement is pushed out of the steel cylinder. Thus, the contact area between the cement and the cylinder will decrease, and hence the total push-out force will decrease.

The curves shown in Figure 6 and Figure 7 were used as inputs for the finite element simulation using the maximum recorded force and its corresponding displacement value. Table 4 shows the results of the debonding simulations, indicating a higher relative error for the room-temperature modelling.

**Figure 8:**
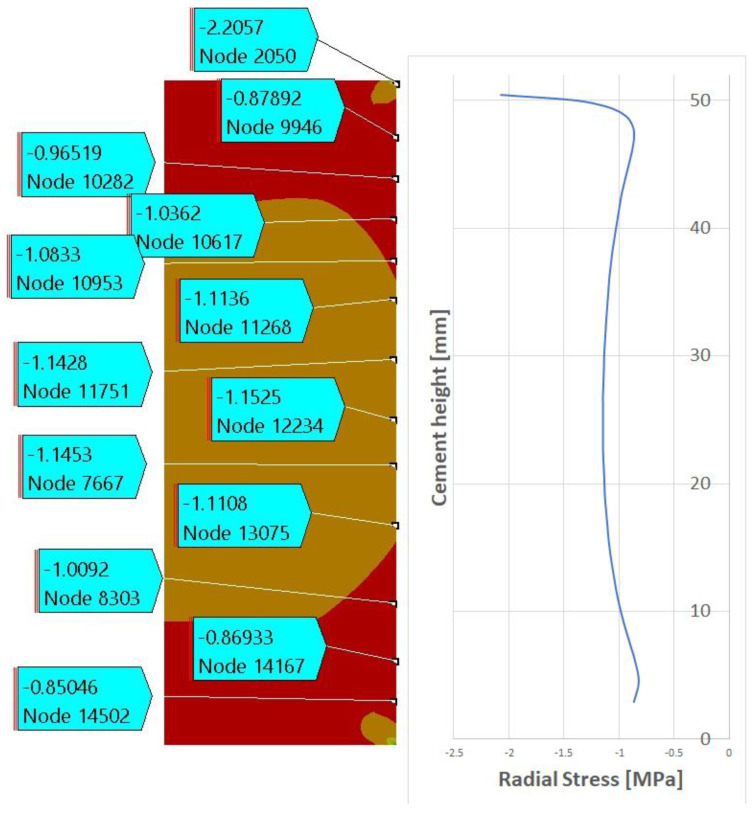
Stress state in the cement after cooling from 75 °C to 30 °C.

Figure 8 shows the stress state inside the model after cooling from 75 °C to 30 °C. As expected, the differences between the metal outer pipe and the cement result in slight compressive stresses at the interface. These stresses are then kept in the model and the debonding is evaluated. 

**Figure 9:**
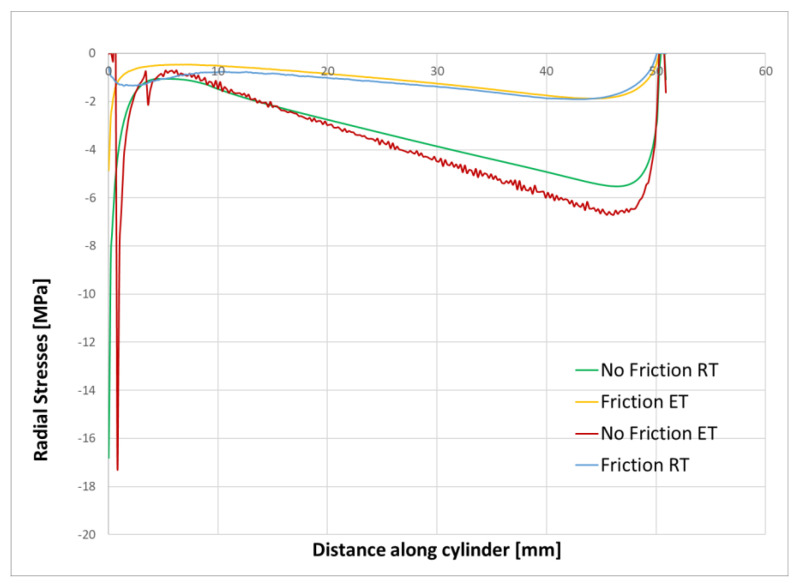
Stress state in the cement samples after 1 day of curing with and without friction considered.

**Figure 10:**
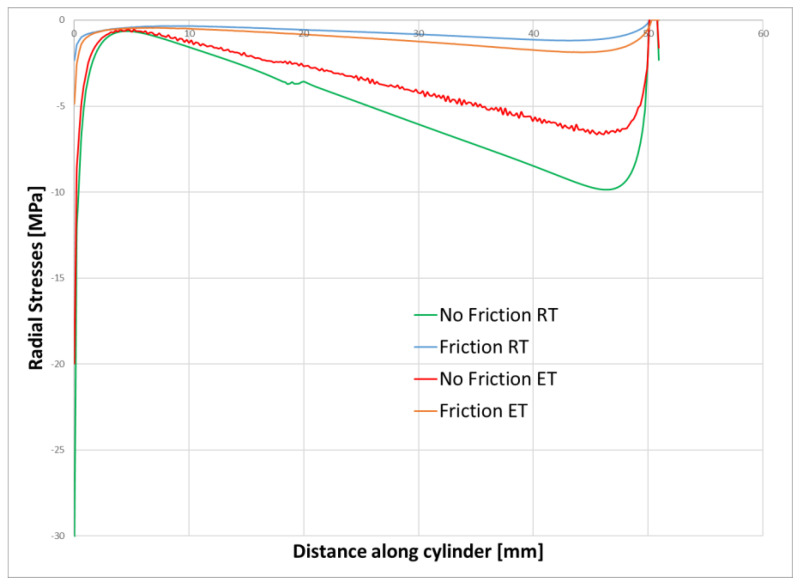
Stress state in the cement samples after 3 days of curing with and without friction considered.

The existence of compressive stresses can explain why, after debonding, the push-out force is not zero, but, rather, the sample behaves as a frictional force between the cement and the steel cylinder. To further investigate this, we extracted from the finite element results the radial contact stresses between the cement and the steel cylinder after debonding. Figure 9 and Figure 10 show the contact stresses at the interface between the cement and the steel cylinder for the 1-day and 3-day samples. The “no friction” curve represents the status of the cement just before debonding, while the “friction” curve represents the status after debonding. We noticed that, post-debonding, a reduction in the radial stress at the cement interface takes place. This is somewhat expected, since there is a significant difference between the bonding of CZM elements and friction-type bonding, as the latter is weaker and thus the resulting stresses will be lower. It seems that the debonding process is the one increasing the stresses in the cement to a level that exceeds the IBSS. Once debonding is lost, the stresses realign at a much lower value (about 30%).

The effect of friction can also be visualized by comparing the debonding failure criteria when friction is considered and when it is not considered. The no-friction scenario represents the debonding situation before the cement plug is pushed down; hence, friction is not considered. After the debonding takes place (modelled using the CZM concept), the bond between the cement and the metal sleeve is considered to be of a frictional nature only, hence it is marked as friction. 

The Mode II-dominated bilinear CZM model we used here assumes that the separation of the materials occurs when the tangential displacement goes beyond a certain value. Technically, a bonded contact is defined as a contact between the two parts. No friction is involved in the process. Basically, when the CZM, defined as a contact between the two bodies, is completely de-bonded, the analysis diverges. At this moment, we make the transition to the frictional contact phase. Our approach was to continue to analyze the cement–pipe contact after debonding by modeling a friction contact. In this case, the cement is slowly moving up to the point where the pushing force is equal to the friction force that develops between the two bodies. According to this model, the cement is no longer kept together with the pipe and the analysis diverges. 

**Figure 11:**
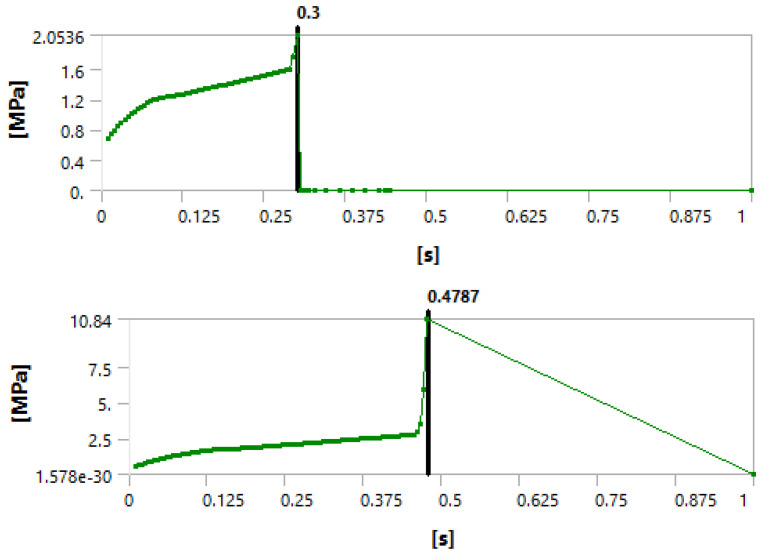
Frictional stress state after 3 days of curing without (**top**) and with (**bottom**) temperature considered.

Figure 11 shows a comparison of the frictional stress state after 3 days of curing, post-debonding (frictional mode only). It can be seen that, for the debonding failure (Figure 11, bottom), a longer time is needed before the sliding of the cement cylinder, which indicates a higher friction force, takes place. This means that the elevated-temperature scenario will inevitably have a higher friction coefficient compared to the lower-temperature scenario.

**Figure 12:**
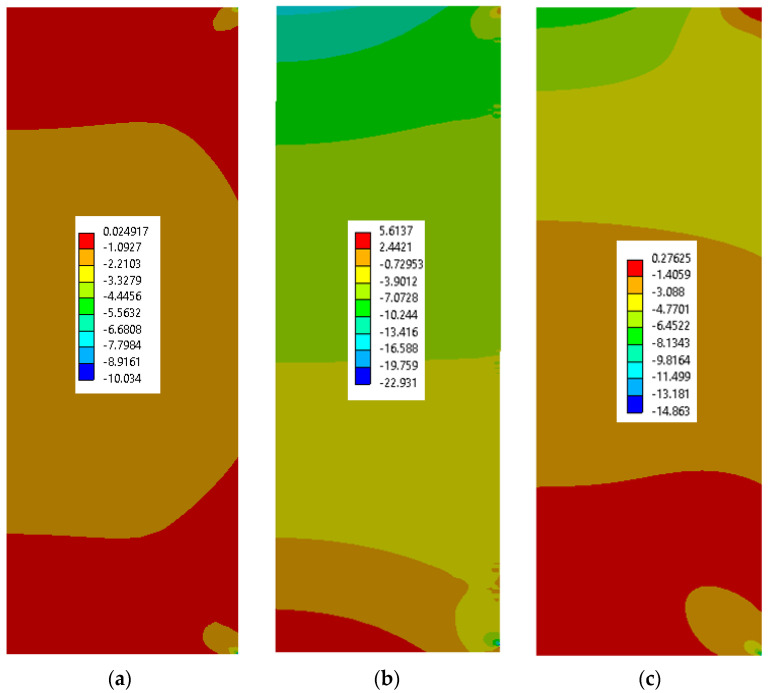
Example of radial stress on the cement plug after cooling (**a**), after debonding (**b**), and after frictional failure (**c**).

**Figure 13:**
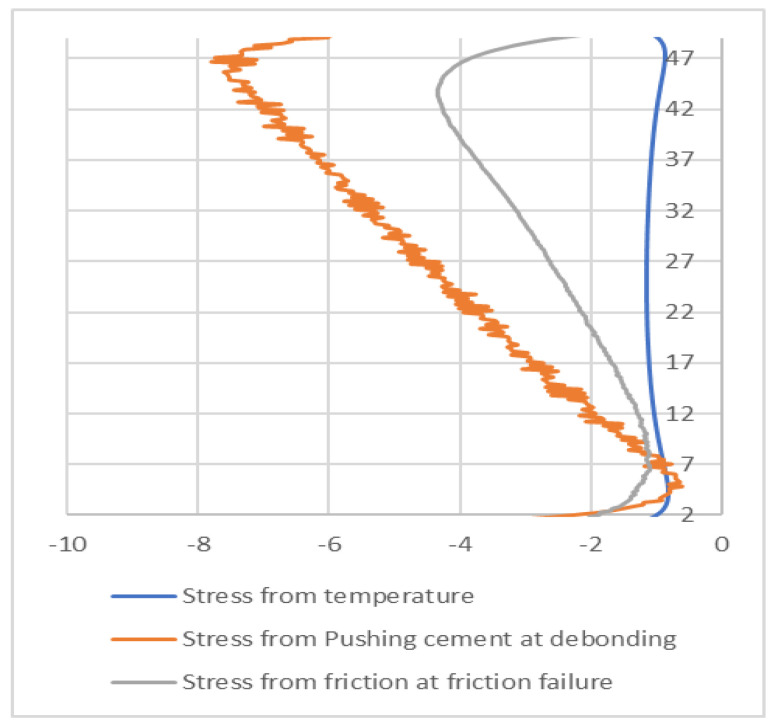
Contact stresses between the cement plug and steel sleeve after cooling (blue), after debonding (orange), and after frictional failure (gray).

Figure 12 shows the radial stress state in the cement plug after the cooling (Figure 12a), debonding (Figure 12b), and friction (Figure 12c) modes. For a detailed understanding, the contact stresses between the cement and the steel sleeve are plotted in Figure 13. It can be seen that the induced stresses after cooling are relatively small, but negative. The highest radial stresses are documented after the debonding failure and, finally, these stresses are reduced during the friction mode. 

After noticing the many errors obtained at RT conditions, as shown in Table 5, we decided to run multiple simulations with varying friction coefficients until a low relative error was obtained. The new results are shown in Table 6. It can be seen that the friction coefficient that leads to low relative errors increases with curing time at RT, while a stabilization is noted after day 7 and almost at day 3 for elevated temperatures. This behavior can be explained by the fact that the hydration of the cement is not complete, especially for the RT tests, and, thus, after debonding, a water film at the interface helps to reduce the friction coefficient. 

## 5. Discussions

This paper aimed to answer two specific questions related to the cement–casing bonding/debonding process, which concerned the influence of elevated temperature and the need for cooling prior to testing and the post-debonding process.

Our simulations showed that, for the debonding process, data obtained during the push-out test will be sufficient to enable finite element modeling of the debonding process using the CZM concept. As an input, we used the experimentally measured IBSS at room temperature and elevated temperature, and both showed good reproducibility with FEM. The cooling of the samples from 75 °C to 30 °C showed little to no effect on the outcomes and the relative error. Thus, we can conclude that, for the proposed samples, geometry, size, and cooling prior to testing do not affect the results.

The most important outcome of our paper, with significant implications for well integrity in real situations, is the post-debonding behavior of the cement. Firstly, we have been able to demonstrate that, after debonding, a given contact stress (radial) still exists between the casing and the cement, thus resulting in the existence of a friction force. This friction force will still hold the casing in place after the debonding has occurred. Additionally, we have noticed that for the RT-cured samples, the friction coefficient may change, showing a constant increase, whereas for the HT tests this increase is much smaller. This difference can be associated with the fact that elevated temperature will accelerate the curing process (hydration), and thus the highest friction coefficient will be achieved faster. The friction coefficient was found to be the same at a curing time of 7 days at both RT and HT. 

Our results will have an impact on well integrity calculations and evaluations. The results show that there is still a casing–holding force post-debonding, which is of a purely frictional nature. This implies that the well may still be mechanically strong (the casing will stay in place); however, debonding may initiate a micro-annulus, and thus leakage will be possible.

## 6. Conclusions

This paper demonstrates that:

1. The cooling of the samples has little to no effect on the IBSS results, but it will affect the post-debonding behavior of the cement–casing system.
2. A new post-debonding model of a casing–cement system using friction bonding elements is proposed.
3. Post-debonding, a friction force between the casing and the cement exists and will affect the relative motion between the casing and the cement.

The following highlights can be found:

- The experimental results were again modeled by FEA with acceptable errors of under 1 to 3% for debonding failure, but with a higher error (2 to 7%) when the casing–cement friction was estimated post-debonding.
- At room temperature, the friction force estimated with FEA was not as expected, with the resulting error exceeding 30 to 40% for a constant friction coefficient. However, accepting that the friction coefficient varies with curing time, the errors will be less than 7%.
- Higher temperature modeling showed very strong correlations. It is assumed that at room temperature, since the cement hydration is not completed within the first 3 days, the cement–casing bonding is adhesive rather than frictional, thus resulting in the higher error. Data suggest that compressive stress induced by cooling will slightly affect the results in the direction that the observed data are higher than the real values. However, it is expected that the opposite results (lower values) should be observed in the steel samples, where the cement is poured outside of the steel pipe, as in Evans’s [5] model.

## Figures and Tables

**Table 1:** Bonding cell geometry, after [6].

Item	Cell Length (mm)	Outer Diameter (OD) (mm)	Inner Diameter (ID) (mm)
Bonding Cell	50	40	35.1

**Table 2:** Interfacial bonding shear strength (IBSS) for the cement samples cured at 1, 3, and 7-days.

Curing Time [Days]	Measured Force RT [N]	Calculated IBSS RT [MPa]	Measured Force 75 °C [N]	Calculated IBSS 75 °C [MPa]
1	15,854	2.798	20,210	3.567
3	37,141	6.556	23,442	4.138
7	28,875	5.097	9372	1.654

**Table 3:** Mechanical properties for the steel and cement materials used for the study.

Material	Young’s Modulus (MPa)	Poisson Ratio (-)
Cement	9000	0.3
Steel	210,000	0.3

**Table 4:** Measured and calculated forces for 1, 3, and 7 days.

Curing Days	Measured Force [N]	Calculated Force [N]	Relative Error [%]
1 day @ RT	15,854	16,300	2.81
3 days @ RT	37,141	38,000	2.31
7 days @ RT	28,875	29,700	2.86%
1 @ 75 °C	20,210	20,490	1.38
3 days @ 75 °C	23,442	23,790	1.48
7 days @ 75 °C	9372	9840	4.99%

**Table 5:** Measured and calculated forces for 1 day and 3 days showing post-debonding forces (F2).

Curing Days	F2 [N]	Calculated F2 [N]	Relative Error [%]
1 day @ RT	2300	2450	28.26
3 days @ RT	10,500	9800	−42.86
1 @ 75 °C	7186	7470	3.95
3 days @ 75 °C	9682	9500	−1.88

**Table 6:** Measured and calculated forces for 1 day, 3 days, and 7 days showing post-debonding forces (F2) and the adopted friction coefficient.

Curing Days	F2 [N]	Friction Coefficient Used for Calculation	Calculated F2 [N]	Relative Error [%]
1 day @ RT	2300	0.3	2450	6.52
3 days @ RT	10,500	0.5	9800	−6.67
7 days @ RT	12,648	0.68	12,500	−1.17
1 @ 75 °C	7186	0.6	7470	1.59
3 days @ 75 °C	9682	0.65	9500	−2.91
7 days @ 75 °C	4743	0.68	4570	−3.65

## Data Availability

Not applicable.
